# The Role of the Immune Response in the Development of Medication-Related Osteonecrosis of the Jaw

**DOI:** 10.3389/fimmu.2021.606043

**Published:** 2021-02-25

**Authors:** Weidong Zhang, Ling Gao, Wenhao Ren, Shaoming Li, Jingjing Zheng, Shasha Li, Chunmiao Jiang, Shuying Yang, Keqian Zhi

**Affiliations:** ^1^Department of Oral and Maxillofacial Surgery, The Affiliated Hospital of Qingdao University, Qingdao, China; ^2^School of Stomatology of Qingdao University, Qingdao, China; ^3^Key Laboratory of Oral Clinical Medicine, The Affiliated Hospital of Qingdao University, Qingdao, China; ^4^Department of Endodontics, The Affiliated Hospital of Qingdao University, Qingdao, China; ^5^Department of Stomatology, Binzhou People'Hospital, Binzhou, China; ^6^Department of Orthodontics, The Affiliated Hospital of Qingdao University, Qingdao, China; ^7^Department of Anatomy and Cell Biology, School of Dental Medicine, University of Pennsylvania, Philadelphia, PA, United States

**Keywords:** jaw, bisphosphonates, osteonecrosis, immunity, inflammation

## Abstract

Medication-related osteonecrosis of the jaw (MRONJ) is a rare but serious adverse drug effect. There are multiple hypotheses to explain the development of MRONJ. Reduced bone remodeling and infection or inflammation are considered central to the pathogenesis of MRONJ. In recent years, increasing evidence has shown that bisphosphonates (BPs)-mediated immunity dysfunction is associated with the pathophysiology of MRONJ. In a healthy state, mucosal immunity provides the first line of protection against pathogens and oral mucosal immune cells defense against potentially invading pathogens by mediating the generation of protective immunoinflammatory responses. In addition, the immune system takes part in the process of bone remodeling and tissue repair. However, the treatment of BPs disturbs the mucosal and osteo immune homeostasis and thus impairs the body's ability to resist infection and repair from injury, thereby adding to the development of MRONJ. Here, we present the current knowledge about immunity dysfunction to shed light on the role of local immune disorder in the development of MRONJ.

## Introduction

Medication-related osteonecrosis of the jaw (MRONJ) is a severe medication-induced necrotic bone disease (1). According to the definition by the American Association of Oral and Maxillofacial Surgeons (AAOMS) in 2014, MRONJ is characterized by the following: “(1) exposed bone in the maxillofacial region that does not heal within 8 weeks after identification; (2) exposure to an anti-resorptive agent; and (3) no history of radiation therapy to the craniofacial region” (2). However, based on newest recommendations (2020) on MRONJ, MRONJ is defined as an “adverse drug reaction described as the progressive destruction and death of bone that affects the mandible and maxilla of patients exposed to the treatment with medications known to increase the risk of disease, in the absence of a previous radiation treatment” (3). Additionally, the workshop of the European task force on MRONJ in 2019 suggested that it may no longer be necessary to require an 8-week observation of potential MRONJ manifestation to fit the case definition (4).

MRONJ was first found in patients receiving anti-resorptive therapy (bisphosphonates) and has not only affected the patients' quality of life but also interfered with the decision-making process of most dentists. Exposure to anti-resorptive medication was identified as a main risk factor of MRONJ. Although dosage and duration of administration were considered to be related to the occurrence of MRONJ (5, 6), recent clinical data from a 14-year retrospective survey showed that the number of patients treated with low-dose anti-resorptive medication accounted for over half of the MRONJ cases (7). In addition, some potential risk factors, including periodontal diseases, tooth extraction or dental implantation, and chemotherapy were reported to be associated with the occurrence of MRONJ (8–11). With aging, the number of people with skeletal-related events (SREs) who need anti-resorptive treatment increases. To balance the risks and benefits of these medications, it is imperative to explore the pathophysiology of MRONJ.

Now, there has been an increase in new kinds of medication that have been reported to induce the occurrence of MRONJ, such as another anti-resorptive medication, a monoclonal antibody against the receptor activator of nuclear factor-κB (RANK) ligand (RANKL): denosumab and vascular endothelial growth factor inhibitors (12): bevacizumab, Sunitinib. The mechanisms of MRONJ differ from medication to medication. The long-term administration of bisphosphonates and denosumab will cause disturbed remodeling due to their effect on osteoclast differentiation. Whereas, bevacizumab and Sunitinib- induced MRONJ may be associated with inhibition of angiogenesis. Currently, findings regarding MRONJ pathogenesis were categorized into several hypotheses, including disturbed bone remodeling, inflammation or infection, altered immunity, soft tissue toxicity, and inhibition of angiogenesis (13). Although accumulating evidence has shown that immunity dysfunction is highly involved in the development of MRONJ (14), there are few reports to systematically demonstrate the role of altered immunity in the development of MRONJ. This review highlights the current knowledge regarding the role of immunity dysfunction in the development of MRONJ, including mucosal and osteo immune responses.

## The Mucosal Immune Response and MRONJ

The mucosal immune system defenses against a variety of microbes and maintains the immune homeostasis in the oral cavity under healthy conditions (15). It has a sophisticated anatomical structure, several indigenous microorganisms, and immune cells. A tightly interlaced cell-to-cell network of epithelial cells acts as a physical barrier, thereby defending external stimuli and balancing the intricate interaction between the host and exterior environment (16). Furthermore, several indigenous microorganisms act as a biological barrier to prevent pathogen colonization (17). Moreover, various immune cells and secreted inflammatory cytokines play an important role in immune surveillance and homeostasis (18, 19). Unfortunately, BPs and risk factors that impact the mucosal immune system, coupled with bacterial infection contribute to the development of MRONJ.

### The Destruction of Mucosal Barrier Protection

**Figure 1:**
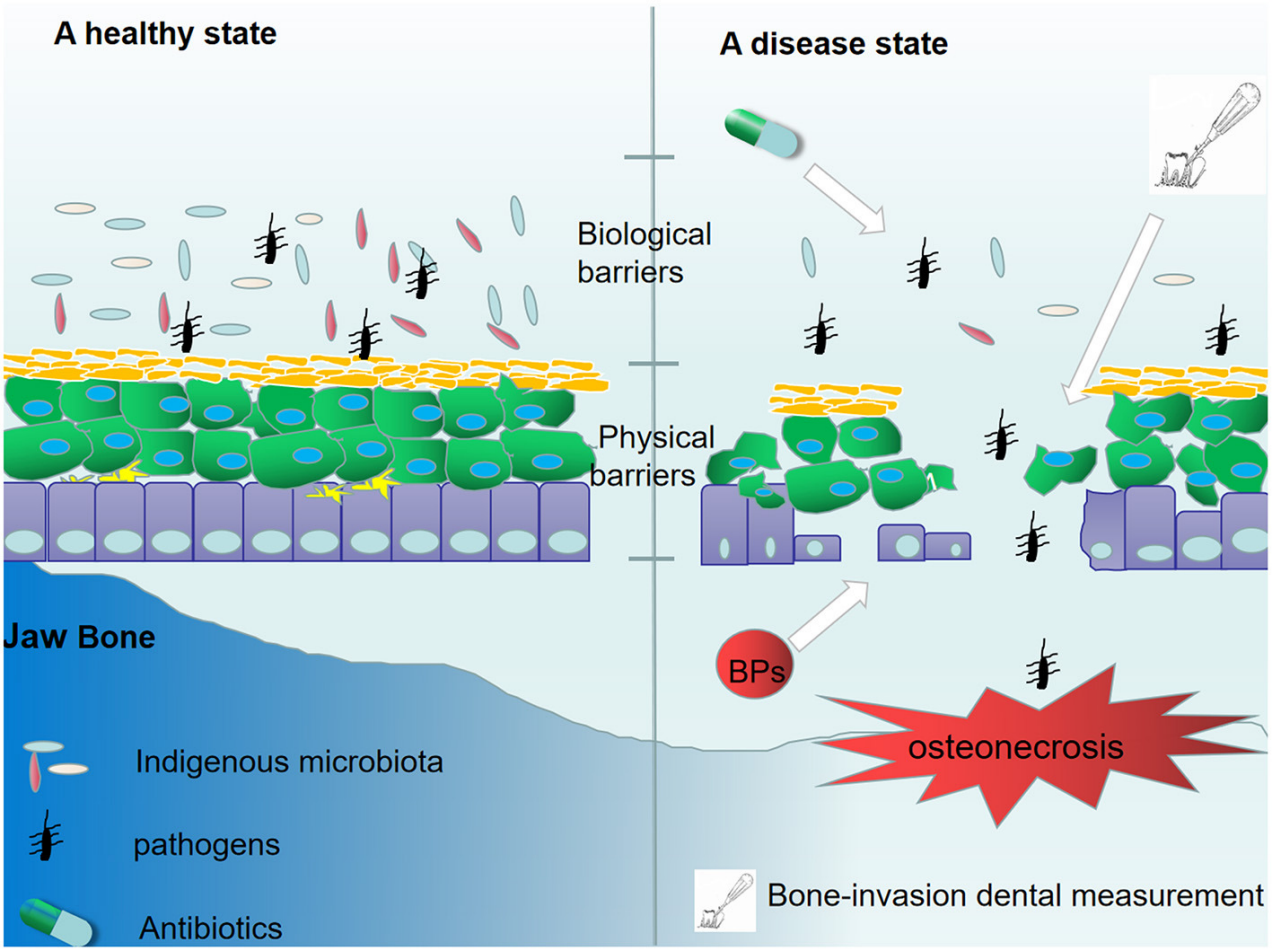
The destruction of mucosal barrier protection. In a healthy state, indigenous microbiota and epithelial cells suppressed the invasion of pathogens. However, the long-time administration of antibiotics disturbs the oral normal flora, leading to a loss of biological barriers. Additionally, treatment with BPs and some bone-invasion dental measurements destroy the physical barrier composed by epithelial cells. The destruction of mucosal barrier protection facilitates bacterial invasion and colonization.

There are hundreds of thousands of bacterial species in the oral cavity (20). An imbalanced bacterial flora can contribute to multiple mucosal diseases (21). Colonization of unique bacterial communities, coupled with a dysfunctional innate immunity, has been shown to affect the pathogenesis of ONJ (22). Indigenous microbiota, as biological barriers, are antagonistic bacteria, which suppress the invasion and colonization of harmful microorganisms. Several protective mechanisms of indigenous microbiota have been reported, including competition for nutrients, direct killing, and enhancement of immune responses (23). In a previous report, it was demonstrated that BPs exerted inhibitory effects on the growth of select bacterial species (24). Recently, Williams et al. demonstrated increased local bacterial infiltration in MRONJ mice (25). Moreover, after extracting healthy teeth from mice, and following with zoledronate infusions, no differences were observed in the formation of osteonecrosis between mice receiving broad-spectrum antibiotic treatment and negative controls. Interestingly, antibiotic-mediated oral dysbiosis causes increased bone necrosis when extracting teeth with ligature-induced periodontitis. Moreover, they showed that broad-spectrum antibiotic treatment suppressed the normal flora to protect against inflammation-induced osteonecrosis by dampening the formation of osteonecrosis and by activating osteoclasts (25). The results showed that the imbalance in oral flora may be a prerequisite for MRONJ and biological barriers are of great necessity to prevent the occurrence of MRONJ (Figure 1). Clinical data also supported the view by the evidence that about half of individuals diagnosed with MRONJ are multiple myeloma (MM) patients, who mostly underwent antibiotics treatment for a long time (26). Although antibiotic treatment has been shown to be effective for MRONJ, increased attention should be paid to patients receiving long-term antibiotics treatment.

Different from the intestinal mucosal barrier, the specific structure of the dento-gingival junction makes the oral mucosal barrier more fragile. The bone-invasion dental measurement and mechanical trauma disrupt the integrity of the oral mucosal epithelial barrier and thus facilitate bacterial invasion and colonization, resulting in jawbone infection. Additionally, mucosal ulceration and periodontal diseases can have a destructive effect on the oral mucosal barrier, and mucosal ulceration is believed to be the initial pathologic event of ONJ (27). BPs have a direct toxic effect on soft tissue (28, 29). Keratinocytes and fibroblasts are two of the most important compartments in the mucosal barrier. Previous reports have shown that BPs can induce senescence of human oral keratinocytes (30) and suppress the cell viability and migration of keratinocytes and fibroblasts (31). Furthermore, high levels of bisphosphonates can cause apoptosis and necrosis (32). Therefore, disruption of the oral mucosal barrier is not only induced by potential risk factors but also by the toxic effect of BPs on soft tissue (Figure 1).

### BPs-Mediated Dysfunction of Immune Cells and Unbalanced Inflammatory Cytokines

Once bacteria break down the mucosal barrier and invade, the hosts' innate immune response is triggered following inflammation to restrict pathogens and sustain homeostasis (33). Inflammation, a manifestation of the body against infection, is characterized by persistent infiltration of immune cells and enhanced levels of multiple pro-inflammatory cytokines and chemokines (34). Inflammation has been confirmed to be a pathological characteristic of MRONJ (35). In several mouse models of MRONJ and clinical ONJ cases, previous studies have shown increased bacterial infiltration and inflammation at the necrotic site (36). BPs, on the one hand, can induce an immunosuppressive condition by suppressing immune cell activation, on the other hand, it can render an imbalance between anti-inflammatory and pro-inflammatory cytokines, thereby resulting in intense inflammation and tissue damage.

#### Dendritic Cells

Dendritic cells (DCs), a type of antigen-presenting cell, play a vital role in initiating and regulating the immune response. Although a limited number of DCs (Langerhans cells) are present in the mucosal immune system, they can contact the external environment and regulate homeostasis of the oral immunity (37). Differentiated from hematopoietic stem cells, DCs are activated by antigens or other factors and present antigens to T cells. Moreover, DCs, as an essential link between innate and adaptive immune responses, are pivotal to maintain mucosal homeostasis (38). Dysfunctional DCs were found to induce an immunosuppressive condition in different diseases (39, 40). In a previous study, it was indicated that BPs exert inhibitory effects on DC activation, leading to immunosuppression or infectious complications (41). Orsini et al. demonstrated that BPs modulate the maturation of DCs but have no effect on the differentiation of DCs (42). Recently, it was shown that zoledronate (nitrogen-containing BPs) treatment decreased the number of infiltrated DCs in soft tissues and impaired the phagocytosis of DCs, thereby leading to an increased bacterial load and the occurrence of MRONJ (43). There are few reports on the underlying mechanism of BPs-mediated dysfunction of DCs, however, the immune suppression and inflammation in the oral cavity caused by dysfunctional DCs may provide new insights into the pathogenesis of MRONJ.

#### Macrophages

Macrophages are a type of heterogeneous mononuclear phagocytes positioned in different tissues, broadly including tissue-resident macrophages and infiltrating inflammatory macrophages (44). Macrophages play key roles in immune surveillance and maintenance of the mucosal microenvironment homeostasis (45). In a clinical experimental study, a lower ratio of CD68/CD14 (CD14 and CD68 are markers of monocytes/macrophages) was observed in MRONJ lesions compared with other jaw infections, thereby indicating that macrophage immunosuppression induced by BPs may be linked to the development of MRONJ (46). Previously, tissue-resident macrophages were viewed as differentiated monocytes that migrated to tissues, however recently, the new view suggested that tissue-resident macrophages are locally being self-renewed (47). Due to the poor self-proliferative capability and short survival time, intestinal macrophages in mucosal immunity were believed to be derived from the constant recruitment of peripheral monocytes (48, 49). Therefore, macrophage migration is crucial for their role in mucosal immunity. However, macrophage migration and morphology were found to be altered by BPs in a dose-dependent manner (50). Lymphatic vessels and blood are required for the migration of immune cells. In a recent report, it was shown that BPs decreased the number of the larger-size F4/80^+^LYVE-1^+^ tube-like-structured cells (a type of macrophage) in impaired socket lesions with inhibition of lymphangiogenesis, which presented new immunopathological features in the MRONJ lesion (51). BPs were reported to suppress the viability of monocytic THP-1 cells after macrophage differentiation (52). The impaired migration and viability of macrophages may be associated with the development of MRONJ; however, there is little direct evidence about affecting the incidence of MRONJ.

**Figure 2:**
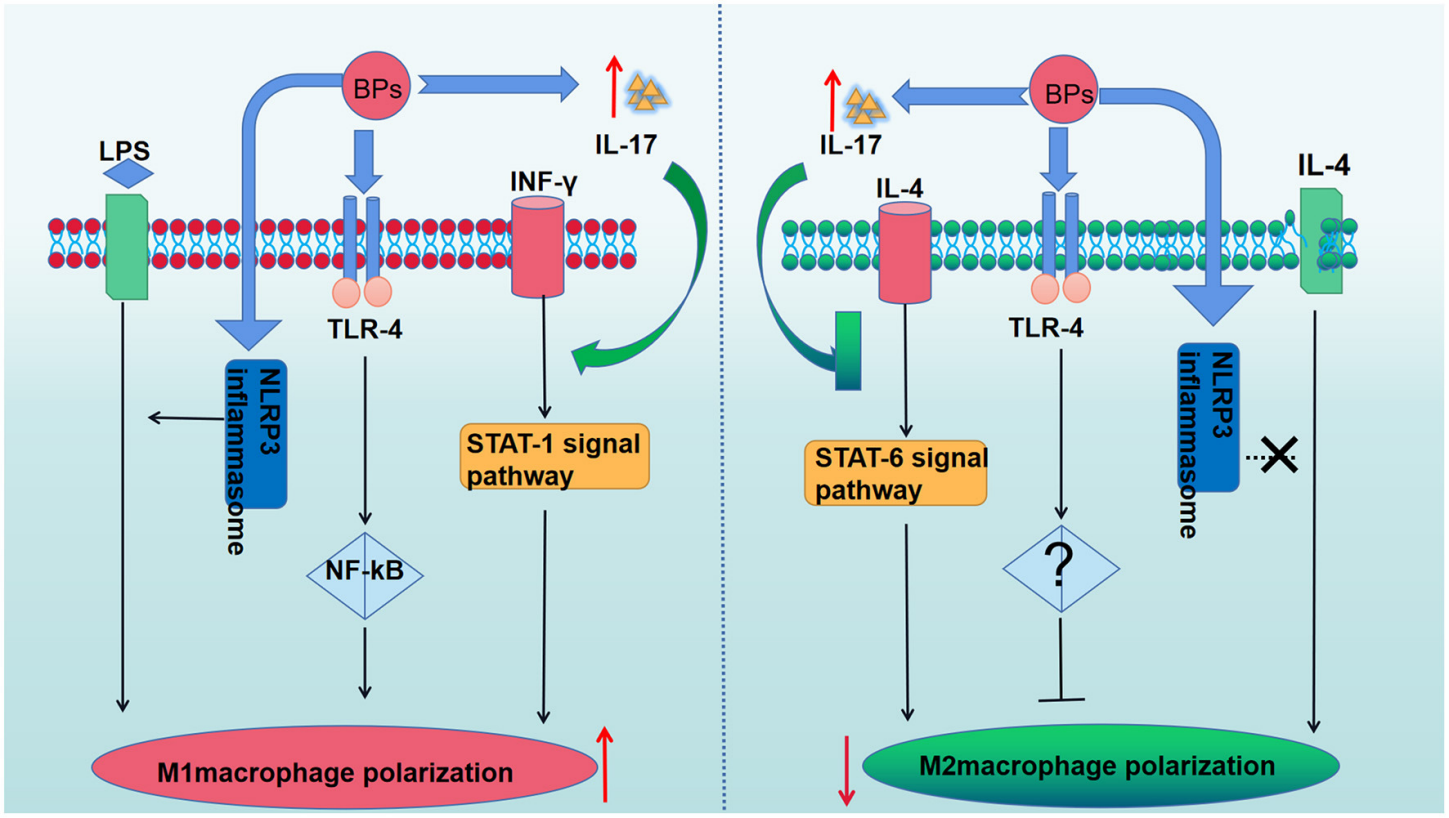
The role of BPs in the macrophage polarization. BPs increase M1 macrophages polarization by activating the TLR-4 signaling pathway and its downstream NF-κB signaling pathway. The polarization of M2 macrophages was suppressed due to the activation of the TLR-4 signaling pathway. BPs enhance LPS-induced M1 macrophages through NLRP3 inflammasome activation but have no effect on the differentiation of M2 macrophages in IL-4-treated human macrophages. The enhanced IL-17 can promote the polarization of M1 macrophages by activation of the STAT-1 signal pathway but inhibit M2 macrophages polarization by affecting IL-4-mediated activation of the STAT-6 signal pathway.

The macrophage phenotype has been shown to play an important role in mediating inflammatory changes in tissues (53). Macrophages can be divided into two phenotypes (M1 and M2 macrophages) (54). Upon the stimulation of T-helper (T_H_) 1 cytokines, such as IFN-γ and lipopolysccharide (LPS), macrophages are converted into M1 macrophages (55). M1 macrophages can produce pro-inflammatory cytokines, nitric oxide (NO), reactive oxygen species (ROS), interleukin (IL)-12, and tumor necrosis factor (TNF)-α, thereby playing an important role in host defense against pathogens. In addition, M2 macrophages are anti-inflammatory and serve an important role in eliminating inflammation and tissue modeling by secretion of IL-10 and TNF-β. M2 macrophages are polarized by stimulation of T_H_2 cytokines, such as IL-4 or IL-13 (55). The balance of M1/M2 macrophage polarization maintains the immune homeostasis and governs the fate of organs. If the infection is serious enough to impair an organ, the M1 phenotype is exhibited to release pro-inflammatory cytokines against the stimulus. However, if the M1 phase persists, the tissue will be destroyed. Therefore, M2 macrophages release anti-inflammatory cytokines to suppress the inflammation and retain homeostasis (54). In recent studies, it was reported that imbalanced polarization of macrophages was associated with the pathogenesis of MRONJ (56). BPs were believed to enhance LPS-induced M1 macrophages through NLRP3 inflammasome activation, thereby amplifying inflammation by secreting pro-inflammatory cytokines (Table 1). Interestingly, it was further demonstrated that zoledronate did not have an effect on the differentiation of M2 macrophages in IL-4-treated human macrophages (THP-1 cells), and zoledronate did not pose an anti-inflammatory role via the polarization of M2 macrophages (65). Zhang et al. found that enhanced T-helper (T_H_)17 cells coupled with IL-17 cytokines correlated with an elevated M1/M2 macrophages ratio at the local mucosal site of MRONJ lesion and that the inhibition of IL-17 activity reduced the elevated M1/M2 macrophages ratio as well as the incidence of MRONJ-like lesions in mice with multiple myeloma. Mechanistically, it was found that IL-17 can promote the polarization of M1 macrophages by activation of the STAT-1 signal pathway but inhibit M2 macrophage polarization by affecting IL-4-mediated activation of the STAT-6 signal pathway (61). In addition, Toll-like receptor family (TLRs)-mediated polarization of macrophages was reported to take part in the development of MRONJ. TLRs belong to pattern recognition receptors (PRRs). There are 12 TLR members. Some TLRs, including TLR-1,−2,−4,−5,−6, and−10, are cell membrane receptors, while others (TLR-3,−7,−8,−9,−11, and−13) are located within intracellular compartments (66). TLR-4 stands out in the polarization of macrophages (57). By inhibiting the mevalonate signaling pathway, zoledronate was reported to activate the TLR-4 signaling pathway and its downstream NF-κB signaling, which then enhanced M1 but inhibited M2 macrophage polarization. Furthermore, blocking the TLR-4 signaling pathway significantly inhibited the effect of zoledronate-mediated polarization of macrophages and decreased the number of M1 macrophages in the extraction socket tissues and the incidence of MRONJ (57). Therefore, TLR-4-mediated macrophage polarization and TLR-4 signaling might present novel therapeutic strategies for the treatment of patients with MRONJ (Figure 2). Taken together, the BPs-mediated alteration in M1/M2 macrophages in the mucosal immune system contributed to the pathogenesis of MRONJ.

**Table 1:** The role of various cell cytokines in the development of MRONJ.

**Cytokine**	**The role and function**	**Reference**
IL-1	Pro-inflammatory factors / Delaying the wound healing	(57, 58)
IL-4	Anti-osteoclastogenic cytokines	(59)
IL-6	Osteoclastogenic cytokines	(59)
IL-10	Anti-inflammatory factor / Anti-osteoclastogenic cytokines	(59, 60)
IL-12	Pro-inflammatory factors	(60)
IL-17	Pro-inflammatory factors / T_H_1 cytokines Osteoclastogenic cytokines	(57, 61) (59)
IL-23	Osteoclastogenic cytokines	(59)
IL-36	Pro-inflammatory factors	(62)
TNF-α	Pro-inflammatory factors	(60)
TNF-β	Anti-inflammatory factor	(60)
IFN-γ	Pro-inflammatory factors / Osteoclastogenic cytokines	(57, 59)
PDGF-BB	Pro-angiogenic and osteogenic cytokines	(63)
sSema4D	Pro-inflammatory factors	(64)

#### γδT Cells

T cell receptors (TCRs) are unique antigen receptors of T cells (67). There are about 10^∧^5 TCRs on the surface of a T cell. According to the different types of TCRs, T cells can be divided into αβT cells and γδT cells (68). It is well-known that αβT cells mainly participate in adaptive immunity, while γδT cells are involved in innate immunity. Moreover, γδT cells exist in the basal layer of oral mucosa and participate in protecting the integrity of the mucosal immune system. They also play a significant role in immunosurveillance, tissue repair, and homeostasis (69). Although BPs were believed to stimulate the expansion and cytotoxic activity of human γδT cells and play a role in cancer immunotherapy (70), in a previous study, it was shown that patients with MRONJ have a deficiency in circulating γδT cells in peripheral blood (71). Human γδ T cells are innate lymphocytes and are mainly composed of Vγ9Vδ2 T cells (71). BPs inhibit osteoclastic bone resorption by suppression of a key enzyme, farnesyl pyrophosphate synthase (FPPS), via the mevalonate pathway isoprenoid synthesis. As the natural substrate of FPPS, isopentenylpyrophosphate (IPP) is an endogenous antigen, which is recognized by a Vγ9Vδ2 T cell. The consequence of BPs-mediated inhibition of FPPS is the accumulation of cellular IPP, which results in Vγ9Vδ2 T cell stimulation in individuals receiving BPs therapy. The repeated activation of Vγ9Vδ2 T cells eventually causes γδ T cell deficiency (72). The loss of Vγ9Vδ2 T cells induced by BPs is defined as activation-induced cell death (73), which is a relatively common regulatory mechanism induced by the immune system to maintain homeostasis (74). Recently, Kalyan et al. found that BPs-treated neutrophils inhibited Vγ9Vδ2 T cell proliferation and that neutrophil-mediated inhibition of γδ T cell expansion could be rescued by suppressing neutrophil-derived hydrogen peroxide, serine proteases, and arginase I activity (75). These findings showed that treatment with BPs has an inhibitory effect on γδ T cells by affecting the function of neutrophils. These results can present reasons for BP-induced γδT cell deficiency, an underlying susceptibility to the development of MRONJ. However, in a recent report, Alexandru, et al. found increased infiltration of γδT cells and elevated expression of soluble semaphorin 4D (sSema4D) in MRONJ lesions whereas γδT cells only accounted for 2–5% of lymphocytes in the blood. SSema4D produced by γδT cells is involved in eliciting inflammation by promoting macrophages to produce pro-inflammatory cytokines, such as TNF-α, IL-1β, and IFN-γ. Consistently, the ablation of γδT cells in mice or neutralization of Sema4D by anti-Sema4D-mAb significantly decreased the incidence of MRONJ (64). Thus, γδT cells may likely be the source of Th1 cytokines, which provides insight into the pathogenesis of MRONJ. However, some researchers observed the incidence of ONJ-like lesions was lower in BPs-treated γδT null cells compared to wild mice. Human-γδT cells were injected to BPs-treated immunodeficient mice, and resulted in oral epithelial hyperplasia, but not osteonecrosis (73). These results illustrated that γδT cells are unlikely to affect the core osteonecrosis mechanism but may serve as an important modifier contributing to the occurrence of ONJ.

#### Neutrophil Granulocytes

Neutrophil granulocytes mainly exist in peripheral blood and take part in the early stage of immune responses (76). Neutrophil granulocytes, lymphocytes, and macrophages arise from hematopoietic stem cells and can differentiate into osteoclasts. Neutrophils are innate immune cells possessing phagocytotic ability and exert immune effects via activation of multiple effector responses and the generation of large amounts of reactive oxygen species (ROS) (77). These cells are also the targets of BPs. Hence, these cells might be of utmost importance in the etiology of MRONJ. Indeed, BPs have been shown to have a pro-inflammatory effect by affecting neutrophil function (78). Additionally, in a study regarding the immune cellular profile, it was shown that the number of polymorphonuclear neutrophils was increased in rats treated with BPs as well as several pro-inflammatory cytokines such as TNF-α and IL-1β, inducible nitric oxide synthase (iNOS), nuclear factor-kappa B (NF-kβ), and IL-18 binding protein (IL-18 bp) (79). These results indicated that BPs-altered neutrophils could exacerbate local inflammatory responses. However, Kuiper et al. suggested that exposure to BPs inhibited neutrophil chemotaxis, neutrophil NADPH oxidase activity, and decreased circulating neutrophils. The recruitment of neutrophils was also confirmed to be suppressed *in vivo*. Although the *in vitro* differentiation of neutrophils was not affected, their life span was shortened by BPs. Together, these results suggested that BPs have the potential to suppress the innate immune system, which possibly contributed to the pathogenesis of MRONJ (80). Moreover, a compromise in neutrophil function induced by BPs may be a potential biomarker for MRONJ susceptibility (81). The BPs-altered neutrophil functional defects may shed light on the role of local immunity in the development of MRONJ.

### The Mucosal Immune Response and the Impaired Wound Healing

MRONJ is characterized by the delayed epithelial wound healing and exposed necrotic bone (1). The mucosal immune system exerts an important effect on controlling infection, as well as wound healing. Keratinocytes and fibroblasts are two of the most important components in the mucosal barrier. High viability of keratinocytes and fibroblasts is essential for epithelial wound healing. BPs have been reported to have a direct toxic effect on soft tissue (82). In addition to the direct toxic effect, BPs also exert an inhibitory effect on wound healing by altering immune cytokines (83). In a previous report, it was demonstrated that higher concentrations of anti-resorptives impaired wound healing accompanied by increased levels of inflammatory cytokines (84). IL-36α, a pro-inflammatory cytokine, is released by epithelial cells or innate immune cells and inhibits TGFβ-mediated collagen expression by activating the extracellular signal-regulated kinase (ERK) signal pathway in mouse gingival mesenchymal stem cells (62). A notably enhanced level of IL-36α and decreased level of collagen were observed in MRONJ lesions. Inhibition of the IL-36α pathway in mice alleviated the development of MRONJ lesions by rescuing the expression of collagen (62). Zhang et al. demonstrated that BPs-mediated activation of pyrin domain-containing protein 3 (NLRP3) in macrophages delayed oral wound healing and contributed to MRONJ-like lesions by the secretion of IL-1β in diabetic mice. Mechanistically, it was found that supplementation with intermediate metabolites of the mevalonate pathway robustly abolished the promotion of BPs on the release of IL-1β from macrophages in response to NLRP3 activation. Together, these results suggested that BP-mediated immune-inflammatory responses impaired socket wound healing and made oral wound susceptible to the development of MRONJ (58). In addition, dynamic polarization shifting from M1 to M2 macrophages plays an important role in wound healing (85). Recently, epidermal growth factor (EGF) was reported to partially rescue the effects of BPs on human oral keratinocytes (HOKs) and human umbilical vein endothelial cells (HUVECs) via the EGFR/AKT/PI3K signaling pathway *in vitro* (86). Additionally, exposure to BPs and bacterial infection at tooth extraction sites decreases the expression of keratinocyte growth factor (KGF), an epithelial cell-specific growth and differentiation factor, and, in turn, the reduction of KGF delays the epithelial wound-healing process, thereby contributing to the development of MRONJ (87). Impaired recruitment of endothelial progenitor cells (EPCs) by BPs was also considered an etiological factor of delayed wound healing. Local injection of EPCs stimulated the healing of MRONJ-like lesions by increasing vascularization and improving epithelial and fibroblast functions (88). However, Yamashita et al. suggested that BPs have an effect on impaired osseous wound healing by decreasing the expression of VEGF-C and MMP-13 but was not associated with angiogenic markers (CD31 and VEGF-A) in the bone marrow or soft tissue wound healing (89). In other words, zoledronate selectively suppressed bone healing but did not influence soft tissue healing in the oral cavity. Osseous wound healing and soft tissue wound healing are two important stages of bone necrosis healing that need further exploration.

## The Osteo Immune Response and MRONJ

Bone is dynamic tissue undergoing continuous remodeling and repair to maintain bone homeostasis (90). Bone remodeling is a process, which is related to osteoblasts-mediated osteogenesis and osteoclasts-mediated osteoclastogenesis. During the healthy state, bone resorption and bone formation take place at equal rates to ensure an adequate quantity of bone mass and a healthy functional status (91). However, inflammatory diseases are always accompanied by bone loss in the oral cavity, such as periodontitis (92). Under the inflammatory condition, the immune system not only protects against the invasive pathogens and reduces inflammation, but also maintains the bone homeostasis by removing the damaged and apoptotic tissue and stimulating the bone tissue repair and regeneration, which fostered a novel interdisciplinary field, “osteoimmunology” (93). Moreover, many regulatory molecules are shared by the immune and skeletal system, including cytokines, receptors, signaling molecules, and transcription factors. Additionally, decades of reports have identified that immune cells participate and mediate skeletal homeostasis by releasing multiple types of cytokines (94). Elsayed et al. found that DCs play a significant role in post-extraction homeostasis in alveolar bone (43). Osteal macrophages were reported to locate adjacent to osteoblasts and regulate bone formation, playing diverse roles in skeletal homeostasis (95). However, BPs affect the process of immune and bone interaction, thereby leading to the imbalance of bone homeostasis (96, 97) and low bone turnover. The low bone turnover promotes the accumulation of microdamage in the jaw bone and the opportunity of bacterial colonization, being suggested as a contributor to the development of MRONJ (98). Therefore, understanding the role of BPs-altered osteo response in the process of bone remodeling is critical to our exploration of the pathogenesis of MRONJ.

### RANK/RANKL/OPG Signaling Pathway and Osteoclastogenesis

RANKL, as one of the important molecules, links bone metabolism and immune response. Initially, RANKL was considered an activator of DCs released by activated T cells (99). Recently, in several reports, it was shown that RANKL could induce the differentiation of osteoclast precursors by binding to RANK, a type I membrane protein (100). Osteoprotegerin (OPG) has an inhibitory effect on the function of RANKL by binding to RANKL and preventing its binding to RANK (100). The relative ratio between RANKL and OPG plays an important role in initiating and maintaining osteoclastogenesis, thereby, resulting in bone resorption by osteoclasts, which is central to the remodeling of the jawbone (101). However, Nisio et al. found the increased expression of RANK, RANKL in BPs induced necrotic bone lesions, suggesting that colonizing bacteria that produced lipopolysaccharide could trigger the RANK/RANKL/OPG signaling pathway and enhance osteoclast differentiation and activation (102). It was suggested that osteoclast activation was a protective strategy from the host bone tissue to eliminate the necrotic area and infection.

**Figure 3:**
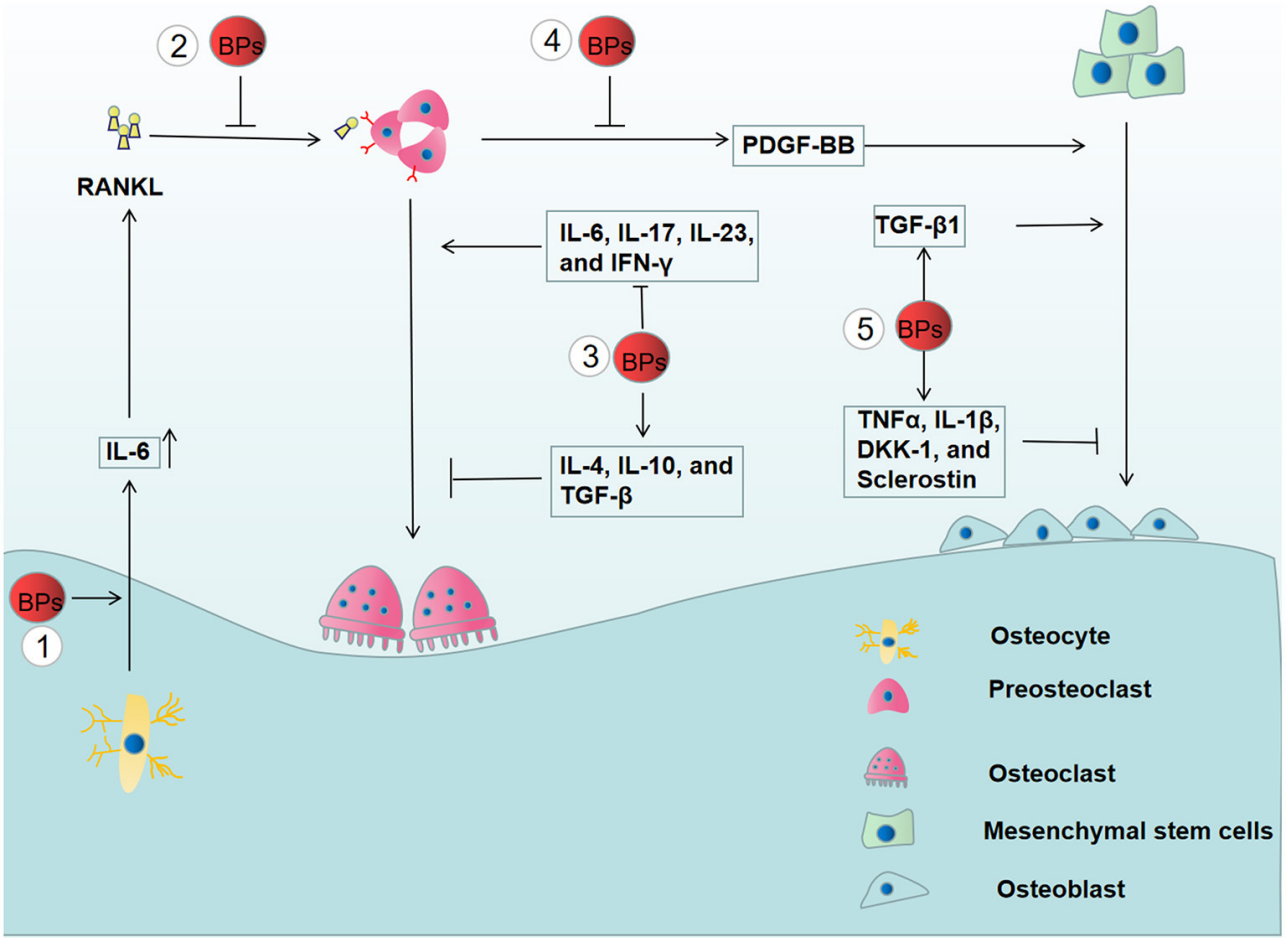
The effects of BPs on bone remodeling. (1) The increased secretion of IL-6 from BPs-treated osteocyte enhances the expression of RANKL, thereby, promoting the osteoclast differentiation. (2) However, BPs inhibit osteoclast differentiation by suppressing the RANKL/OPG/RANK signaling pathway. (3) BPs have an inhibitory effect on the osteoclast differentiation by suppressing the osteoclastogenic cytokines (IL-6, IL-17, IL-23, and IFN-γ) and increasing anti-osteoclastogenic cytokines (IL-4, IL-10, and TGF-β). (4) Osteogenic differentiation is inhibited by the reduced release of PDGF-BB from preosteoclasts. (5) Moreover, BPs affect osteoblastogenesis by altering the release of the pro-inflammatory mediators, including TNFα, IL-1β, DKK-1, sclerostin, and TGF-β1.

In a previous report, it was illustrated that BPs modulate the expression of RANK, RANKL, and OPG, thus decreasing osteoclast activity (103). Moreover, BPs were confirmed to inhibit RANKL-induced osteoclast differentiation by the suppression of the nuclear factor of activated T-cells c1 (NFATc1) (104). As a transcription factor of the nuclear factor in the activated T cell (NFAT) family (105), NFATc1 is a vital downstream target of RANK. When RANKL binds to RANK, TNF receptor-associated factor 6 (Traf6) is recruited and activated, thereby causing signal pathway activation of NF-κB, MAPK, and c-Fos (106). These signal pathways trigger the activation of NFATc1 as well as the process of osteoclast differentiation. These results demonstrated that BPs impaired bone homeostasis by affecting the RANKL/OPG/RANK signaling pathway (Figure 3).

### Cytokines and Osteoclastogenesis

Immune cells not only have the capacity to eliminate inflammation but also affect osteoclast differentiation by the release of multiple cytokines, including osteoclastogenic and anti-osteoclastogenic cytokines (107). Osteoclastogenic cytokines, such as TNF-α, IL-1, IL-6, IL-7, IL-8, IL-11, IL-15, IL-17, IL-23, and IL-34, promote osteoclast differentiation, while anti-osteoclastogenic cytokines have an inhibitory effect on osteoclast differentiation, including IFN-α, IFN-β, IFN-γ, IL-3, IL-4, IL-10, IL-12, IL-27, and IL-33. Clinical data showed that BPs-treated osteoporotic patients have significantly decreased levels of IL-6, IL-17, IL-23, and IFN-γ and a significant increase in IL-4, IL-10, and TGF-β compared to osteoporotic patients who did not receive BPs treatment. These results demonstrated that BPs have an inhibitory effect on osteoclast differentiation by reducing osteoclastogenic cytokines and increasing anti-osteoclastogenic cytokines (59). Recently, it was demonstrated that osteocytes play an important role in inflammatory bone resorption mediated by inflammatory cytokines (108). However, BPs were reported to increase the expression of IL-6 and subsequently trigger elevated RANKL expression in osteocyte-Like MLO-Y4 cells, which potentiates osteoclast formation (109) (Figure 3). Thus, it is necessary to further explore the relationship between the alterations induced by BPs and the development of MRONJ.

### Cytokines and Osteogenesis

Bone remodeling not only requires osteoclasts to eliminate damaged bone tissue but also requires osteogenesis and angiogenesis to form new bone. In a recent study, it was shown that the occurrence of MRONJ was associated with a deficiency in a subset of γδT cells in human peripheral blood. In addition, it was shown that circulating γδT cells played a pivotal role in bone regeneration rather than just inducing an inflammatory response (110). Furthermore, it was demonstrated that γδT cells can stimulate the release of IL-17A to promote bone formation and the healing of fractures (111).

Platelet-derived growth factor-BB (PDGF-BB), secreted by preosteoclasts, was reported to promote angiogenesis and osteogenesis (63). Gao, et al. reported that zoledronate can inhibit the secretion of PDGF-BB from preosteoclasts, and decreased PDGF-BB suppressed the angiogenic function of endothelial progenitor cells and osteoblastic differentiation of mesenchymal stem cells (112). Moreover, it was found that the number of preosteoclasts and PDGF-BB secretions were significantly reduced in BPs-treated rats coupled with decreased numbers of microvessels and osteoblasts in the early stage of bone healing. Recombinant PDGF-BB rescued the proliferative, migration, and osteogenic functions of impaired mandible-derived bone mesenchymal stem cells in MRONJ-like rats. Finally, PDGF-BB has been confirmed to exhibit therapeutic effects on MRONJ-like rats (113). Together, these results illustrated the important role of PDGF-BB in the development of MRONJ (Figure 3).

Experimental data showed that a low dose of BPs significantly increased the expression of genes essential for osteogenic differentiation possibly by upregulating the TGF-β1 production. These findings provided a new view on the development of MRONJ (114). However, Giannasi et al. found that low doses of BPs could inhibit osteoblastogenesis by enhancing the release of the pro-inflammatory mediators, including TNFα, IL-1β, DKK-1, and Sclerostin (115) (Figure 3). Therefore, the role of BPs-mediated osteogenesis in the development of MRONJ is yet not completely understood.

## Conclusions and Future Directions

In this review, we focused on the role of the immune response in the pathogenesis of MRONJ. Treatment with BPs disturbs mucosal immune homeostasis, thereby leading to prolonged oral inflammation and delayed tissue repair. In addition, BPs have an inhibitory effect on the process of bone remodeling in an osteoimmune manner. These results suggested that altered immunity plays an important role in the development of MRONJ. Unfortunately, there are few reports on synergistic or antagonistic effects among various immune cells on the pathogenesis of MRONJ. To better understand the role of the immune response in the development of MRONJ, we should pay more attention to the immune regulation network and the interaction between immune cells and necrosis. Furthermore, current experimental research is predominantly from *ex vivo* studies or animal models and there is a lack of relevant reports on the clinical treatment of MRONJ by immune intervention. It is well-known that there is no significantly effective therapy to cure MRONJ. Recently, there are increasing reports about the application of mucosal vaccination to treat several diseases, such as AIDs (116) and Pertussis (117). Further investigations are required to focus on the treatment of MRONJ using immunotherapy strategies. In addition, it is of great significance to seek excellent biomarkers for early diagnosis and prevention of MRONJ. Several reports have identified that some cytokines are associated with the development of MRONJ. However, the clinical effectiveness and sensitivity of these biomarkers are required to be explored in future studies.

## Author Contributions

WZ, LG, WR, JZ, ShaoL, and ShasL collected the data. WZ and LG wrote the paper and drew the pictures. CJ, SY, and KZ reviewed and edited the paper. All authors contributed to the article and approved the submitted version.

## Conflict of Interest

The authors declare that the research was conducted in the absence of any commercial or financial relationships that could be construed as a potential conflict of interest.
