# Supplementary material for: The Role of the Immune Response in the Development of Medication-Related Osteonecrosis of the Jaw
Source: Front Immunol. 2021 Feb 25;12:606043. doi: 10.3389/fimmu.2021.606043 (PMC7947359; doi:10.3389/fimmu.2021.606043)
Supplement: Supplementary file 1 [file Table_1.DOCX]

Supplementary Material

**Table 1 Role of various cell cytokines in the development of MRONJ**

| **Cytokine Source The role and function Reference** |
| --- |
| IL-12 M1 macrophages Pro-inflammatory factors (*88*)  TNF-α M1 macrophages Pro-inflammatory factors (*88*)  IL-10 M2 macrophages Anti-inflammatory factor (*88*)  TNF-β M2 macrophages Anti-inflammatory factor (*88*)  IL-1 M1 macrophages Pro-inflammatory factors (*46*)  Macrophage Delaying the wound healing (*80*)  IL-17 M1 macrophages Pro-inflammatory factors (*46*)  TH17 Promoting the polarization of M1 macrophages (*44*)  Inhibiting M2 macrophages polarization (*44*)  IL-17A γδT cell Promoting bone formation and bone fracture healing (*86*)  IL-36α epithelial cells Inhibiting the TGF-mediated collagen expression (*81*)  and innate immune cell  PDGF-BB Preosteoclasts Promoting angiogenesis and osteogensis (*75*)  VEGF promoting the biologic function of monocytes (*89*)  and the differentiation of multinuclear osteoclasts (*89*)  Promoting macrophage recruitment and M2 polarization (*90*)  Promoting the activation of macrophages (*91*) |
